# Secretion of a recombinant protein without a signal peptide by the exocrine glands of transgenic rabbits

**DOI:** 10.1371/journal.pone.0187214

**Published:** 2017-10-27

**Authors:** Andrea Kerekes, Orsolya Ivett Hoffmann, Gergely Iski, Nándor Lipták, Elen Gócza, Wilfried A. Kues, Zsuzsanna Bősze, László Hiripi

**Affiliations:** 1 Department of Animal Biotechnology, NARIC-Agricultural Biotechnology Institute, Gödöllö, Hungary; 2 Department of Biotechnology, Friedrich-Loeffler-Institut, Institut für Nutztiergenetik, Mariensee, Neustadt, Germany; Baylor College of Medicine, UNITED STATES

## Abstract

Transgenic rabbits carrying mammary gland specific gene constructs are extensively used for excreting recombinant proteins into the milk. Here, we report refined phenotyping of previously generated Venus transposon-carrying transgenic rabbits with particular emphasis on the secretion of the reporter protein by exocrine glands, such as mammary, salivary, tear and seminal glands. The Sleeping Beauty (SB) transposon transgenic construct contains the Venus fluorophore cDNA, but without a signal peptide for the secretory pathway, driven by the ubiquitous CAGGS (CAG) promoter. Despite the absence of a signal peptide, the fluorophore protein was readily detected in milk, tear, saliva and seminal fluids. The expression pattern was verified by Western blot analysis. Mammary gland epithelial cells of SB-CAG-Venus transgenic lactating does also showed Venus-specific expression by tissue histology and fluorescence microscopy. In summary, the SB-CAG-Venus transgenic rabbits secrete the recombinant protein by different glands. This finding has relevance not only for the understanding of the biological function of exocrine glands, but also for the design of constructs for expression of recombinant proteins in dairy animals.

## Introduction

Sheep β-lactoglobulin [1] and human tissue plasminogen activator [2] were the first proteins produced in the milk of transgenic mice in 1987. Recombinant proteins may be sourced not only from milk, but from blood, egg white, and seminal plasma. Purifying recombinant proteins from milk has several advantages: milk is easily collectable from lactating animals, tremendous amounts of proteins can be harvested, and milk contains only small amounts of proteases. In contrast to milk, bioactive proteins in the blood of transgenic animals may have adverse effects on the animals’ health, and blood samples are more frequently contaminated with pathogens (review [3]).

Transgenic rabbits were also proven to be potential tools for the secretion of human proteins in 1990 [4], and are now considered as resources to produce pharmacologically active proteins in their milk. Ruconest®, a human C1 esterase inhibitor, was pioneering among the milk-borne recombinant proteins; it is now approved by the authorities in the EU and North America for the treatment of patients with hereditary angioedema, and has eventually reached the market (http://www.pharming.com/). We contributed to this field with transgenic rabbit lines, which produced biologically active proteins, e.g. human tissue-nonspecific alkaline phosphatase (TNAP) [5,6], human blood clotting factor VIII [7] and low-phenylalanine kappa-casein [8] under the control of the whey acidic protein gene promoter. Other livestock species (pigs, sheep, goats and cows) are also used to produce recombinant proteins in their milk. In contrast to the above-mentioned species, it is easier to generate transgenic rabbits due to their high fertility, short gestational time and high number of offspring. Moreover, the milk of ruminants may contain prion proteins [9], but rabbits [10] and pigs [11] are insensitive to prion disease.

Besides the mammary gland, other glands have been approached for recombinant protein expression, including salivary, lacrimal and seminal glands. The salivary glands are potential sources for secreting foreign proteins into saliva fluid, especially in mice [12] and pigs [13]. The parotid secretory protein (PSP) promoter [14] exclusively directed expression into saliva, e.g. in beta-glucanase [15] and aflatoxin-detoxifizyme transgenic mice [16]. Lacrimal gland specific transgene expression was reported in rabbits where in vitro gene transfer was performed using transduced cultured lacrimal gland epithelial cells with adenovectors carrying a tumor necrosis factor (TNF)-inhibitor gene [17]. Seminal fluid was also suggested as a suitable source for bioactive peptides, and it was demonstrated that the mouse P12 gene promoter can be used to generate transgenic mice that express human growth hormone in their seminal vesicle epithelium [18].

The common design for expression of a secreted recombinant protein includes a gland-specific promoter driving a cDNA or a genomic coding region, where the first 20 codons represent a signal peptide (SP) for the exocrine pathway.

The recent finding that reporter transposon sows secrete fluorophore reporters in the milk despite the absence of a signal peptide [19] prompted us to ask whether this will be mirrored in the milk of transposon-transgenic rabbits, moreover other glands were included in the analyses. In conclusion, a refined phenotyping in CAG-Venus rabbits was performed with particular emphasis on fluorophore secretion of exocrine glands, such as mammary, salivary, tear and seminal glands.

The Sleeping Beauty (SB) transposon system was applied in our laboratory for the first time to create transgenic rabbits expressing the Venus fluorophore protein [20]. The Venus reporter protein is a yellow-shifted derivative of the commonly used enhanced green fluorescent protein (EGFP). In the established transgenic rabbit line, one monomeric transgene copy expressed the fluorophore protein driven by the CAG promoter at high levels. CAG is a robust, composite promoter, consisting of the CMV immediate early enhancer, the chicken beta-actin promoter and the rabbit beta-globin intron [21]. As predicted by the construct design, a ubiquitous expression with a cytoplasmic localization of the reporter was found in the initial analyses (20). The SB-CAG-Venus rabbits were further characterized for reporter protein expression during spermatogenesis [22].

Here we describe and characterize the secretion of the signal peptide-less Venus reporter by mammary, salivary, tear and epididymal glands of SB-CAG-Venus transgenic rabbits. Secretion of recombinant proteins into the milk of transgenic animals without post-translational modifications (PTMs) may be advantageous for proteins, such as lysostaphin, where PTMs, such as glycosylation lead to inactivation[23].

## Materials and methods

### Animals

SB-CAG-Venus transgenic rabbits and New Zealand White rabbits as control animals were used in this study. New Zealand White rabbits were purchased from S&K-LAP Ltd. (Kartal, Hungary). SB-CAG-Venus transgenic rabbits were produced in our lab using New Zealand White breed from S&K-LAP Ltd. Animals were kept under standard light-dark cycle (06.00–18.00 h) at 19°C with food and water available ad libitum and caged separately. This study was carried out in strict accordance with the recommendations and rules in the Hungarian Code of Practice for the Care and Use of Animals for Scientific Purposes. The protocol was approved by the Animal Care and Ethics Committee of the NARIC-Agricultural Biotechnology Institute and Pest County’s governmental office (permission numbers: PEI/001/329-4/2013; PEI/01/857-3/2015). The method used for euthanasia: concussion under anesthesia (ketamine/xylazine). All efforts were made to minimize suffering.

### Development of the SB-transgenic rabbit

Creation of Venus transposon transgenic rabbits has been published [20,24]. Briefly, the founder rabbits were created by coinjecting the CAG-Venus transgene flanked by the inverted repeats of the SB transposon and the transposase mRNA. The developing embryos were transferred into surrogate mothers and identified by transgene-specific PCR. The founder animal carrying a single copy of Venus was used to establish the transgenic line.

### Sample collection, fractionation and Western blotting

SB-CAG-Venus transgenic does were mated with SB-CAG-Venus transgenic bucks (Table 1). Mothers were transiently separated from their pups, injected with oxytocin (Kela Laboratoria NV, Belgium, 2 IU i.m.) and milked in the second week of lactation.

**Table 1:** Genotypes of the analyzed rabbits.

Doe ID	Transgene	Buck ID	Transgene
#3010	Non-transgenic	#4030	SB-CAG-Venus, Ho
#3013	WAP-hTNAP, HE	#4020	SB-CAG-Venus, Ho
#3014	WAP-hTNAP, HE	#4017	SB-CAG-Venus, HE
#4034	SB-CAG-Venus, HE	#4019	SB-CAG-Venus, Ho
#4035	SB-CAG-Venus, Ho		

Abbreviations: HE: heterozygote, Ho: homozygote, SB: Sleeping Beauty, WAP: whey acid protein promoter, hTNAP: human tissue-nonspecific alkaline phosphatase.

Tear and oral saliva fluid samples were collected from sexually mature (5–6 months) bucks with medical swab sticks. To retrieve the samples the swab sticks were washed with tris buffered saline (TBS). Semen was collected from sexually mature transgenic and control bucks using artificial vagina. Seminal fluid was purified from semen following centrifugation at 3.000 rpm at 4°C for 90 minutes (min).

To remove the fat fraction, milk samples were centrifuged at 13.000 rpm for 30 min at 4°C. The whey and protein fractions were separated by adding 25% acetic acid and centrifuged at 13.000 rpm for 30 min at 4°C. Total protein was isolated from the fractionated samples and the samples were loaded to SDS-PAGE gel.

Samples derived from transgenic and non-transgenic rabbits were examined on 12% SDS-PAGE gels for their residual protein content. Protein content was estimated with Coomassie Brillant Blue-R (PageBlue, Vilnius, Lithuania) and silver staining (Sigma, St. Louis, MO). For Western blotting a polyclonal rabbit anti-eGFP (1:2000, Thermo Fisher Scientific, USA CAB4211) primary antibody was used with a horseradish peroxidase-coupled anti-rabbit secondary antibody (1:10000; Sigma-Aldrich, USA).

Densitometry analysis (ChemiDocMP Imaging System, BioRad) was performed to estimate the amount of Venus protein in the samples.

### Tissue histology and fluorescence microscopy

Mammary gland tissue samples of a SB-CAG-Venus transgenic doe were collected during lactation and fixed in 4% (w/v) formaldehyde (PFA) at 4°1C for 24 h. After incubation in PFA, samples were replaced in 30% (w/v) sucrose-PBS solution and stored until embedding at 4^°^C. Mammary gland samples were embedded into cryomedium before cryosectioning (Cryomatrix, Thermo Fisher Scientific, USA) and cut into 10 μm thick sections on a cryostat (Microm, Heidelberg, Germany). Nuclei were stained with Topro-3-iodide (Thermo Fisher Scientific, USA, T3605). The images of the lactating mammary gland were obtained with a Leica TCS SP8 confocal microscope equipped with a PMT detector. The detection range of the Venus channel was 510–550 nm. Nuclear staining was recorded at 650–726 nm.

## Results

In most cases, mammary gland-specific promoters and regulatory elements were used to express recombinant proteins in the milk of transgenic animals, e.g. the whey acid protein gene promoter in mice [25] and in rabbits [7]; the β-casein gene promoter in cattle [26]; the ovine β-lactoglobulin gene promoter in sheep[27].

**Fig 1:**
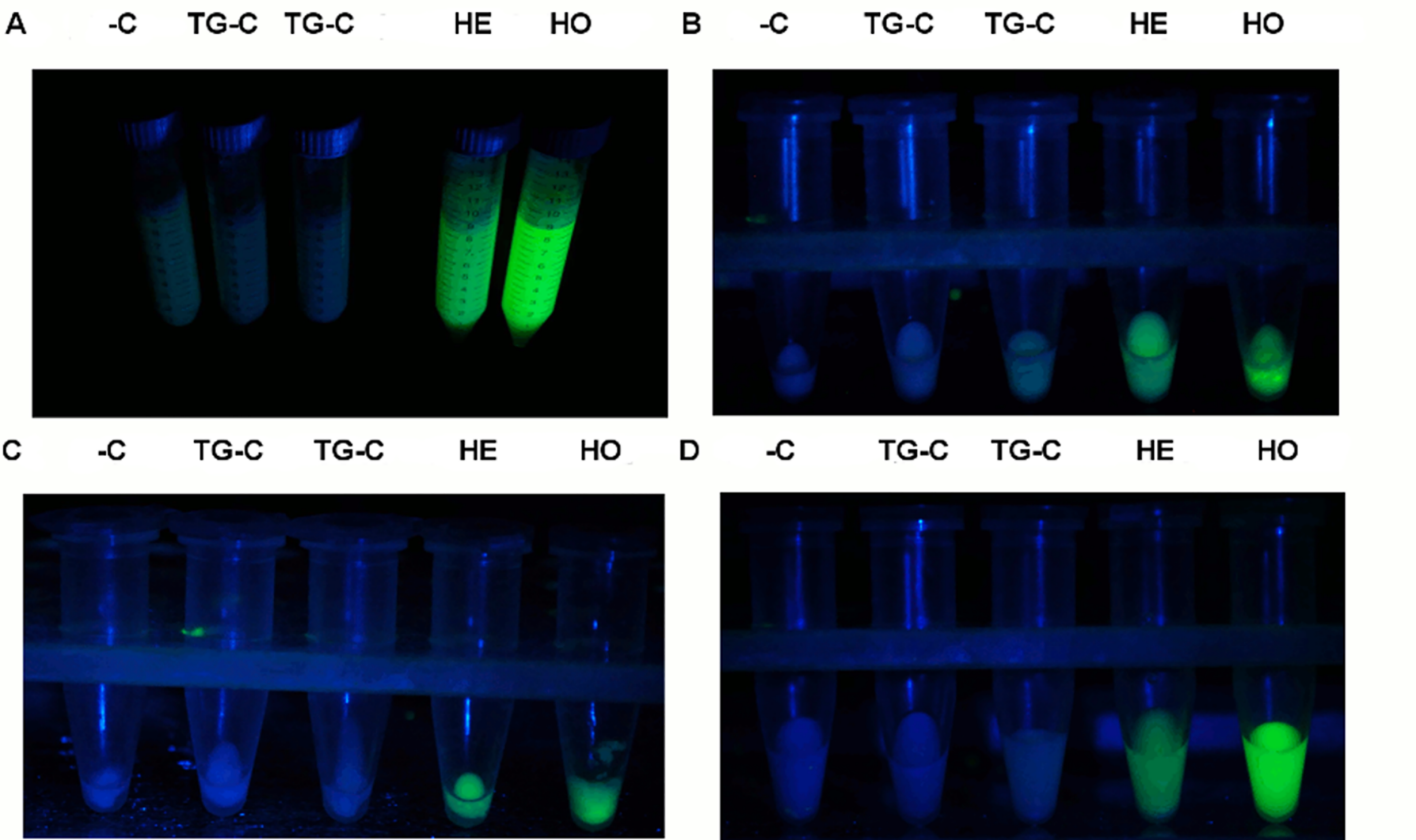
Macroscopic excitation of the Venus fluorescence protein in the milk samples. (A) Venus-specific fluorescence in the whole milk samples and (B) in the fat, (C) milk cell and (D) whey fractions. -C -negative control; TG-C -negative transgenic control; He -Heterozygous; Ho -homozygous.The fluorescence of Venus protein was detected using blue light illumination (FSH/LS-1B) with a barrier filter cutoff below 500 nm with a GFSP-5 headset (BLS, Hungary).

In the present study, we demonstrated the expression of the Venus recombinant protein driven by the ubiquitous CAG promoter in milk and other biological fluids of transposon-carrying transgenic rabbits despite the absence of a signal peptide sequence for the secretory pathway. Milk samples of the SB-CAG-Venus transgenic does (ID numbers: #4034, #4035) showed specific Venus fluorescence, whereas this expression pattern was not observed in milk samples of human TNAP transgenic does (ID numbers: #3014, #3013) and a non-transgenic doe (ID number: #3010) under the same conditions (Fig 1A). As foreign protein expression in milk might modify light absorption and reflection, we used samples from wild-type and a TNAP transgenic line as controls.

The fat and milk cell fractions from both homozygote and heterozygote SB-CAG-Venus transgenic does were Venus positive (Fig 1B and 1C); the strongest Venus fluorescence was found in the whey fraction of the homozygote SB-CAG-Venus transgenic doe (Fig 1D). Venus protein was detected by Western blot analysis in all milk fractions of the SB-CAG-Venus transgenic does during a lactation period. The dynamics of recombinant protein expression was also evaluated. We did not find notable differences between them throughout the lactation period (data not shown).

**Fig 2:**
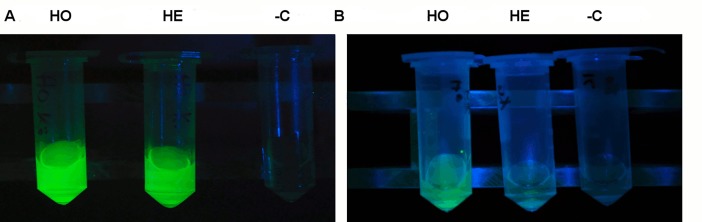
Macroscopic excitation of the Venus fluorescence protein in tear and oral saliva samples. (A) Venus-specific fluorescence in tear and (B) saliva samples. -C -negative control; He -Heterozygous; Ho -homozygous. The same parameters were used to detect the fluorophore protein as described in (Fig 1).

To assess whether the secretion of the signal peptide-less reporter is a unique property of the mammary gland, the fluids of other exocrine glands of the transgenic animals were analyzed. Of the biological fluids of SB-CAG-Venus transgenic rabbits, tear saliva and seminal plasma (Fig 2A and 2B and S2 Fig) were Venus-positive under specific excitation light illumination, whereas samples of wild-type rabbits did not show any specific signal. The tear and oral saliva samples of homozygote bucks showed stronger Venus fluorescence than those of heterozygote bucks, according to the genotype.

**Fig 3:**
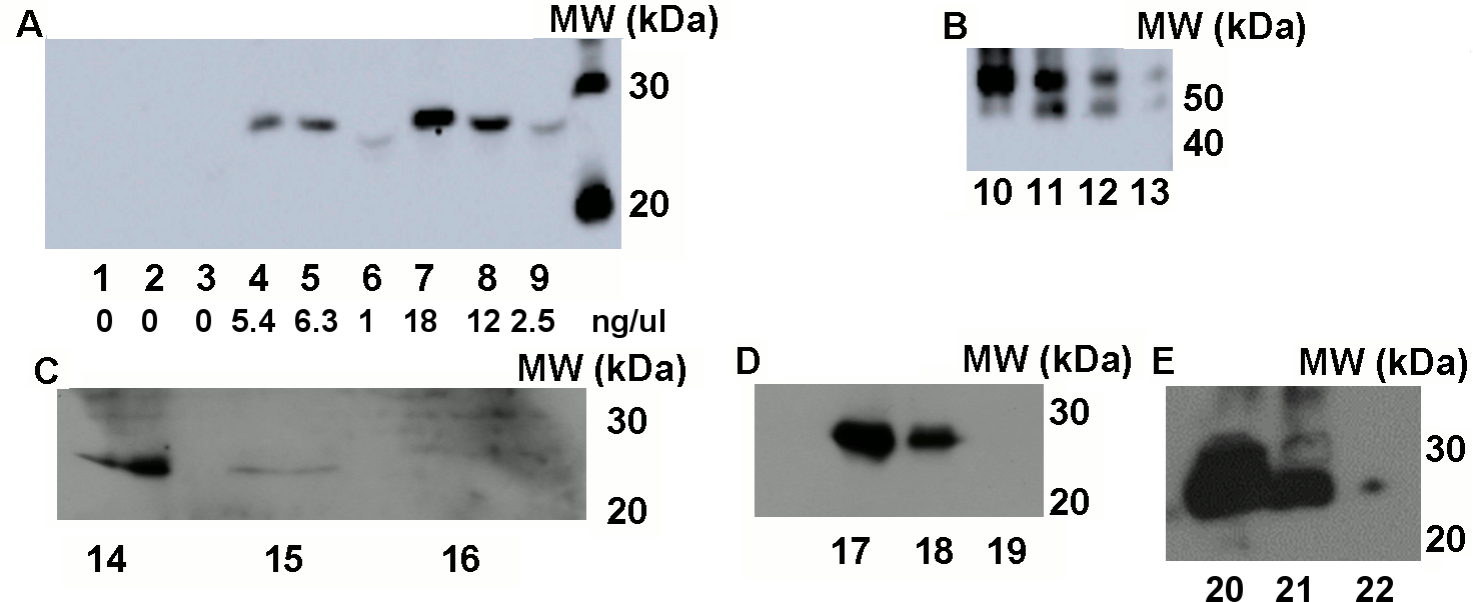
Western blot analysis of milk fractions. (A) Milk fractions of a wild-type doe: 1- Whey fraction, (#3014), 2- Fat fraction, (#3014), 3- Milk cell fraction (#3014);Milk fractions of a heterozygous doe: 4- Whey fraction (#4034), 5- Fat fraction (#4034), 6- Milk cell fraction (#4034);Milk fractions of a homozygous doe: 7- Whey fraction, (#4035), 8- Fat fraction, (#4035), 9- Milk cell fraction (#4035), (20 μg/slot). Calculated recombinant protein concentrations are also given in ng/μl.(B)Dilution series of a recombinant GFP-fusion protein (58 kDa).10–250 ng, 11–125 ng, 12–62.5 ng, 13–31.25 ng. (C)Expression of Venus fluorophore in saliva (D) tear fluid (E) seminal plasma samples of the SB-CAG-Venus homozygote, heterozygote and control bucks. 14, 17, 20: #4020 homozygote, 15, 18, 21: #4017 heterozygote, 16, 19, 22: control buck.

This expression pattern was also verified with Western blot analysis in tear, saliva and seminal fluids (Fig 3C, 3D and 3E)respectively.The concentration of the recombinant protein was determined by UV fluorescence of the different body fluids and confirmed by semiquantitative western blots in heterozygous animals. The concentrations were as follows: seminal plasma 847 ng/μl; whole milk 219 ng/ μl; tear 49 ng/μl, saliva 12 ng/μl.

The largest amount of Venus protein was detected in the milk samples of homozygote transgenic does (compare #4035 to #4034), underlining the transgene dose effect on recombinant protein production. In milk fractions of non-transgenic does no Venus protein was detectable (Fig 3A). To assist efficient evaluation of expression levels, different concentrations of a recombinant GFP-fusion protein (58kDa) were also blotted (shown on Fig 3B). Recombinant protein concentrations were determined in transgenic milk samples using densitometry analysis and presented in (Fig 3A).

**Fig 4:**
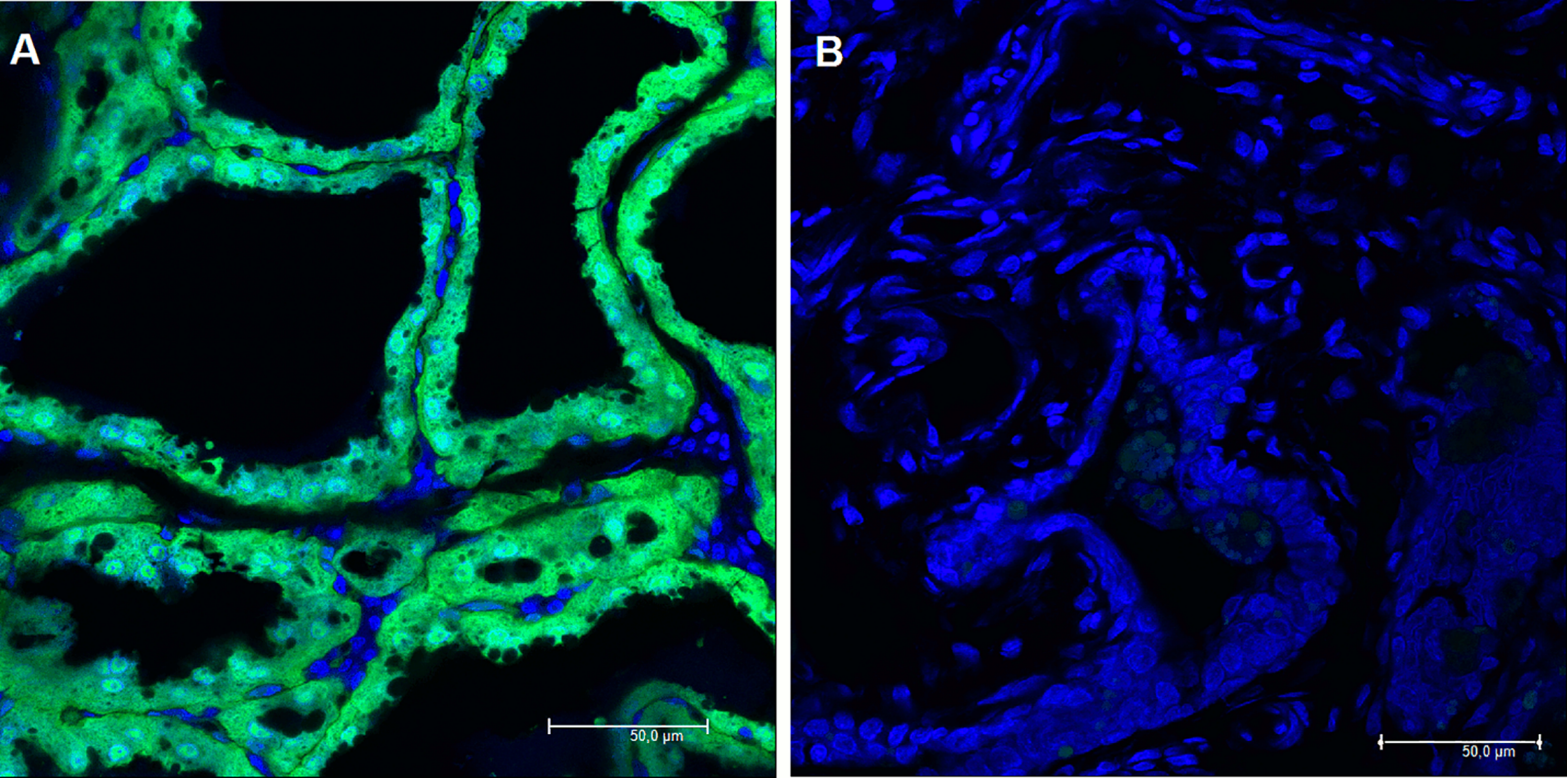
Fluorescence of SB-CAG-Venus lactating transgenic rabbit mammary gland. (A) Tissue section of a heterozygote SB-CAG-Venus transgenic doe showed direct Venus fluorescence at 400X magnification. (B) The section of a non-transgenic doe did not show specific fluorescence under identical conditions. Nuclei were stained with Topro-3-iodide (blue). Scale bars (bottom right) in all images are 50 μm.

Densitometry analysis underlined the macroscopic observations and Western blot analysis: larger amount of Venus fluorophore protein was detected in all fractions of the biological fluids of the homozygote as compared to the heterozygote doe. Sections of mammary gland tissue of a heterozygote SB-CAG-Venus transgenic doe showed cytoplasmically located Venus fluorescence (Fig 4A) as described earlier in other organs [20], whereas the control doe did not express Venus protein (Fig 4B).

Venus expression in the mammary epithelial cells clearly shows that the source of recombinant protein in rabbit milk could be the exfoliated epithelial cells, which reached the end of their secretory life. The distribution of recombinant fluorescent protein in the mammary gland of homozygous females was identical to that of heterozygous animals (S1 Fig). As expected, the tissue and milk samples of the non-transgenic and human TNAP transgenic rabbits remained Venus-negative (Figs 1A–1D and 4B).

## Discussion

In the milk samples of the transgenic pigs generated with the same transgenic construct [28], Venus protein was observed both in whey and milk cell fractions, but only reduced amounts in the fat fractions. In addition, mCherry recombinant protein controlled by the same CAG promoter was obviously enriched in the milk cells [19]. Overall, the phenomenon of signal peptide-less reporter expression pattern in milk samples showed species specific differences, which might be the consequence of the differential total somatic cell counts (SCC) and the cell type distribution in milk [29]. In rabbit the total SCC number is estimated to be 0.5–1x10^6^ cell/ml, similarly to porcine 1x10^6^ cell/ml, out of which 60–90% are epithelial cells [30]. By contrast, the SCC number is 0.075x10^6^ cell/ml in bovine, with very low epithelial cell numbers, and about 61% of macrophages [29], underlining the importance of a careful choice of species.

Although the mammary gland and the prostate are modified apocrine sweat glands, whereas salivary glands (parotid, sublingual, submandibular) and lacrimal glands are merocrine glands [31,32], both types of glands secreted the Venus fluorophore into milk, saliva, seminal and tear fluids, respectively (Figs 1A–1D, 2A, 2B and 3C–3E). The expression of the Venus reporter protein was restricted to the cytoplasm; therefore PTMs (e.g. phosphorylation, glycosylation, lipidation, etc.) could not modify the protein. Importantly, the SB-CAG-Venus construct did not encode a signal peptide [19], thus the Venus protein was not transported via the secretory pathway (endoplasmic reticulum, Golgi apparatus, secretory vesicles). Recombinant protein expression under a mammary gland-specific promoter may result in inappropriate PTMs. For example, the efficiencies of the essential γ-carboxylation of the recombinant human protein C (rhPC) and the recombinant human Factor IX driven by the whey acid protein gene promoter were different at similar expression levels in transgenic pigs [32]. Glycosylation heterogeneity was observed in transgenic goats producing human recombinant antithrombin under the control of the β-casein promoter [33].

In general, imperfect PTMs may impair the effectivity of the desired recombinant proteins secreted into the milk of transgenic animals.

## Conclusions

Our results showed that SB-CAG-Venus transgenic rabbits are able to secrete the Venus reporter protein into different biological fluids, which is another evidence for the robust and ubiquitous expression of a recombinant protein driven by the CAG promoter. Thus if the desired recombinant protein does not require PTMs, expression of its coding region under the tissue-nonspecific CAG promoter could be an attractive alternative for large-scale production. Our results verified that the suggested biotechnological application, namely the cytoplasmic production of recombinant proteins driven by a strong ubiquitous promoter could be feasible in rabbits.

## Supporting information

S1 Fig Fluorescence of SB-CAG-Venus lactating transgenic homozygous rabbit mammary gland. Scale bar is 50 μm (bottom right).
(TIF)

S2 Fig Macroscopic excitation of the Venus fluorescence protein in the seminal plasma. (A) room illumination (B) UV illumination. TG- transgenic, -C- negative control. Note: fluorescent signal could even be seen at room illumination.
(TIF)
