# Supplementary figures and images for: Secretion of a recombinant protein without a signal peptide by the exocrine glands of transgenic rabbits
Source: PLoS One. 2017 Oct 27;12(10):e0187214. doi: 10.1371/journal.pone.0187214 (PMC5659788; doi:10.1371/journal.pone.0187214)

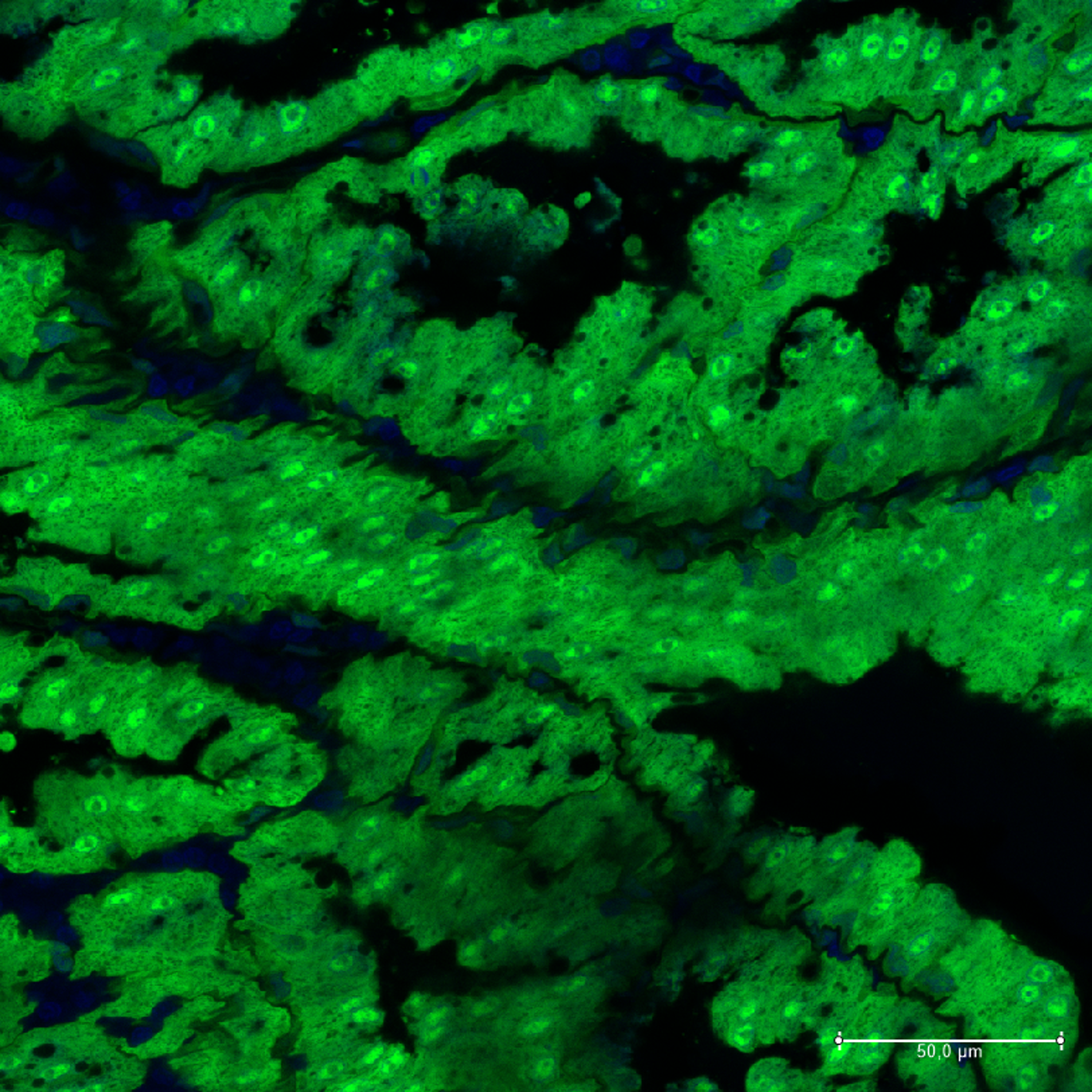

Supplement: S1 Fig — Scale bar is 50 μm (bottom right). (TIF) [file pone.0187214.s001.tif]

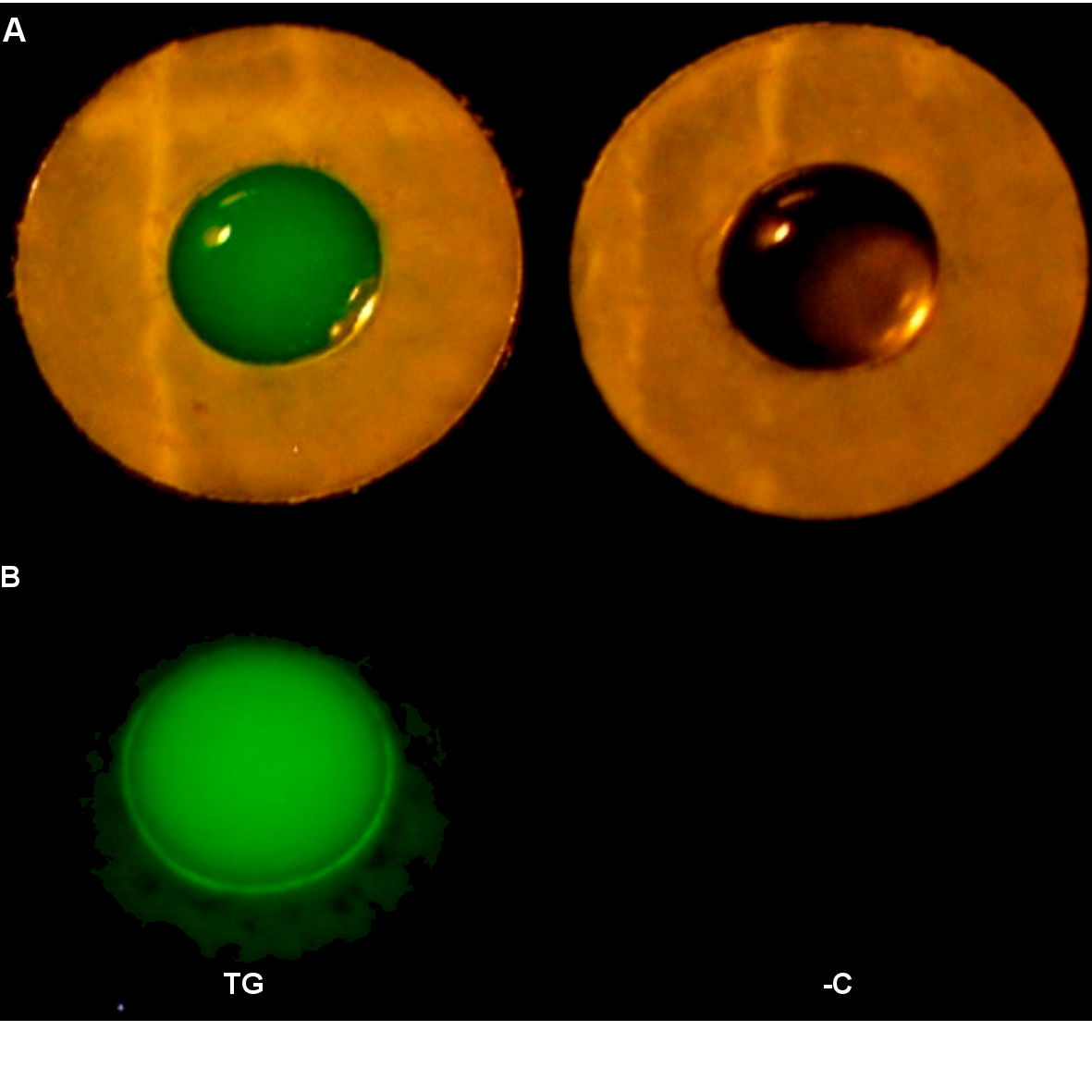

Supplement: S2 Fig — (A) room illumination (B) UV illumination. TG- transgenic, -C- negative control. Note: fluorescent signal could even be seen at room illumination. (TIF) [file pone.0187214.s002.tif]
